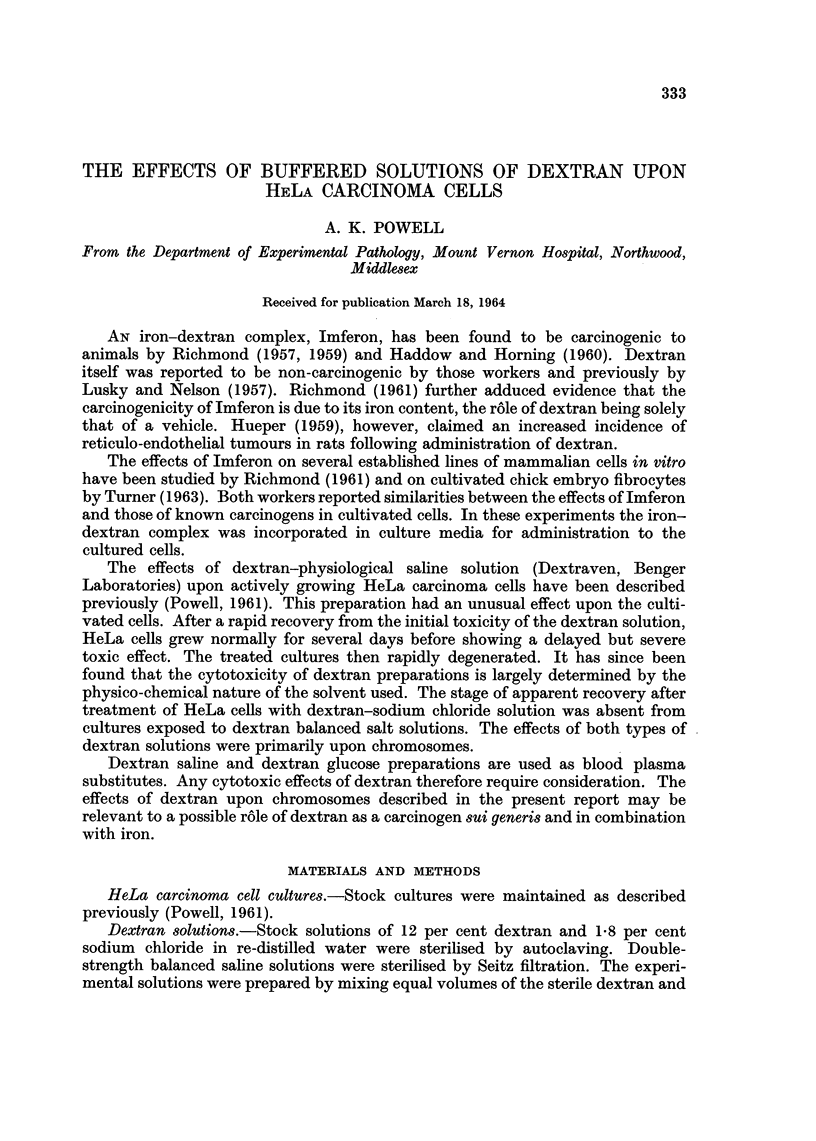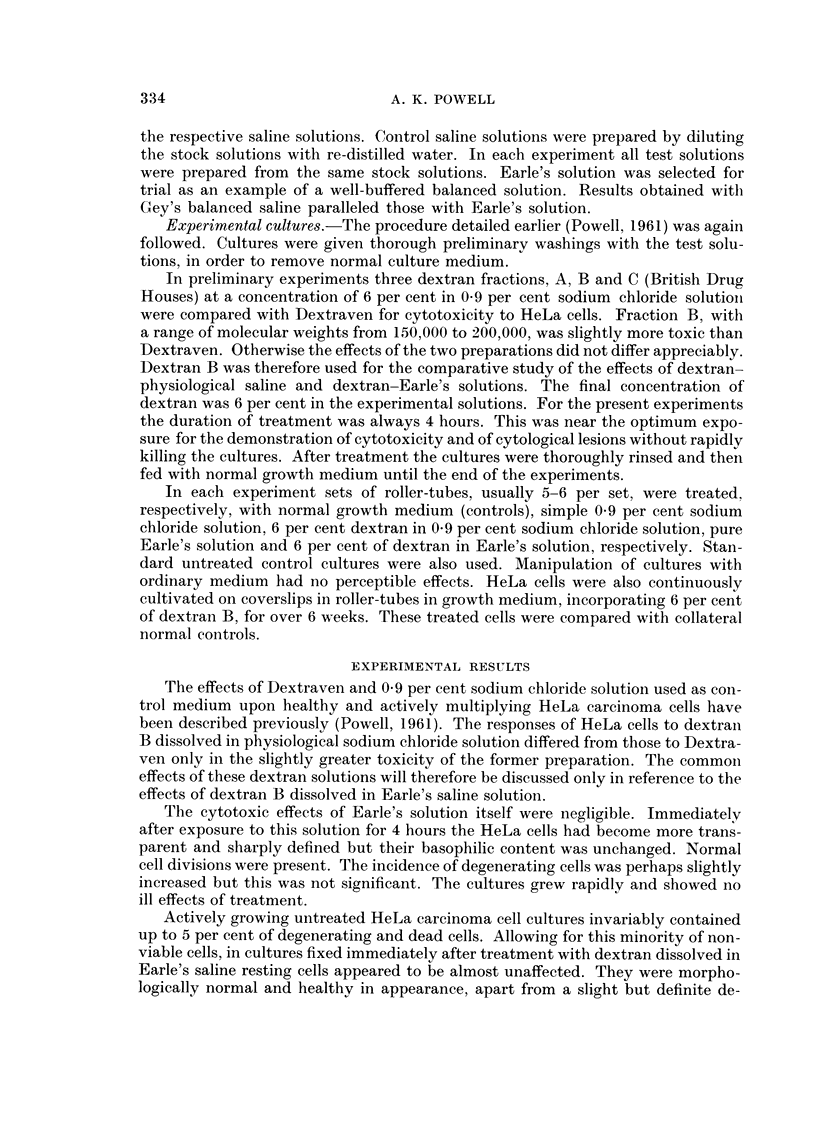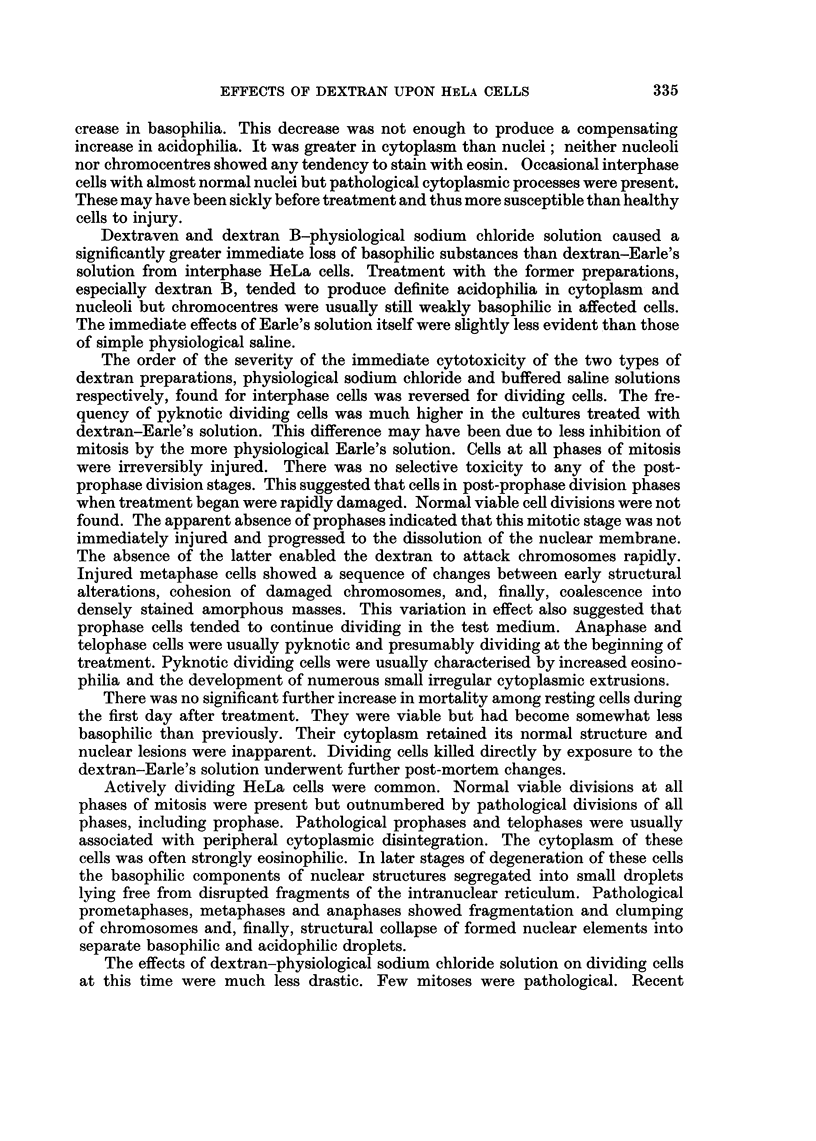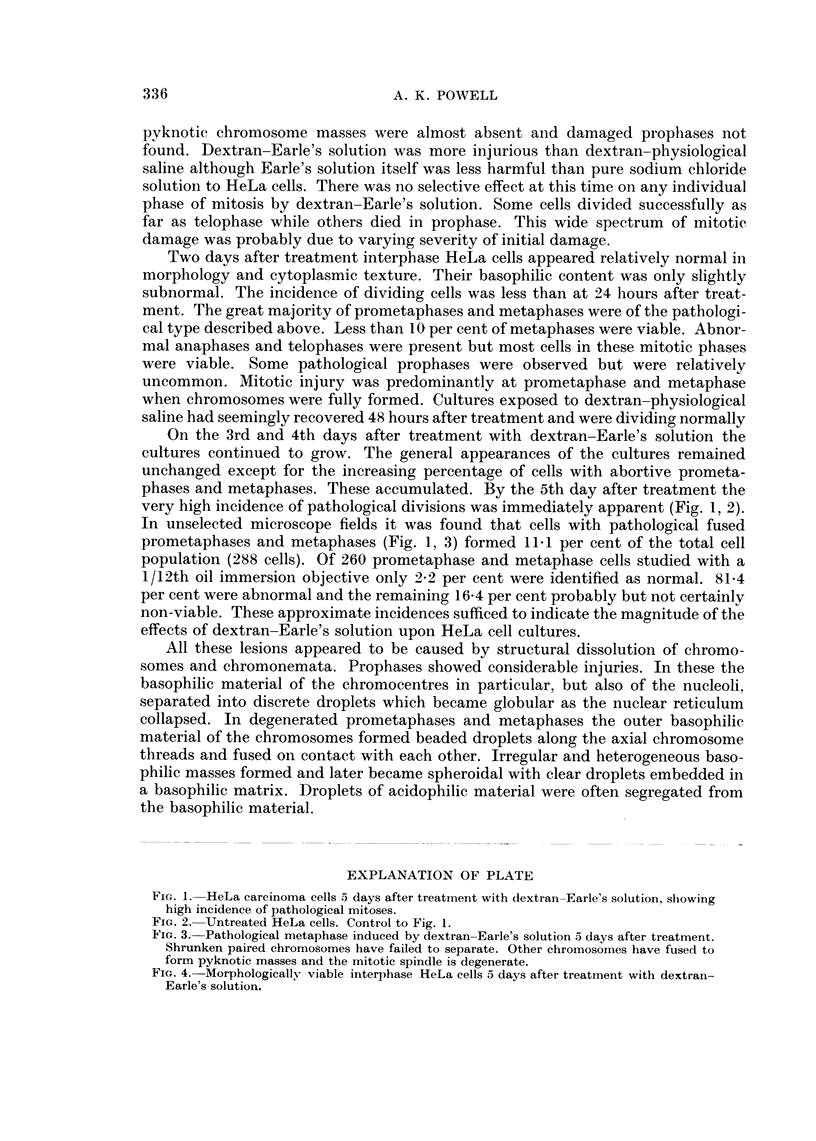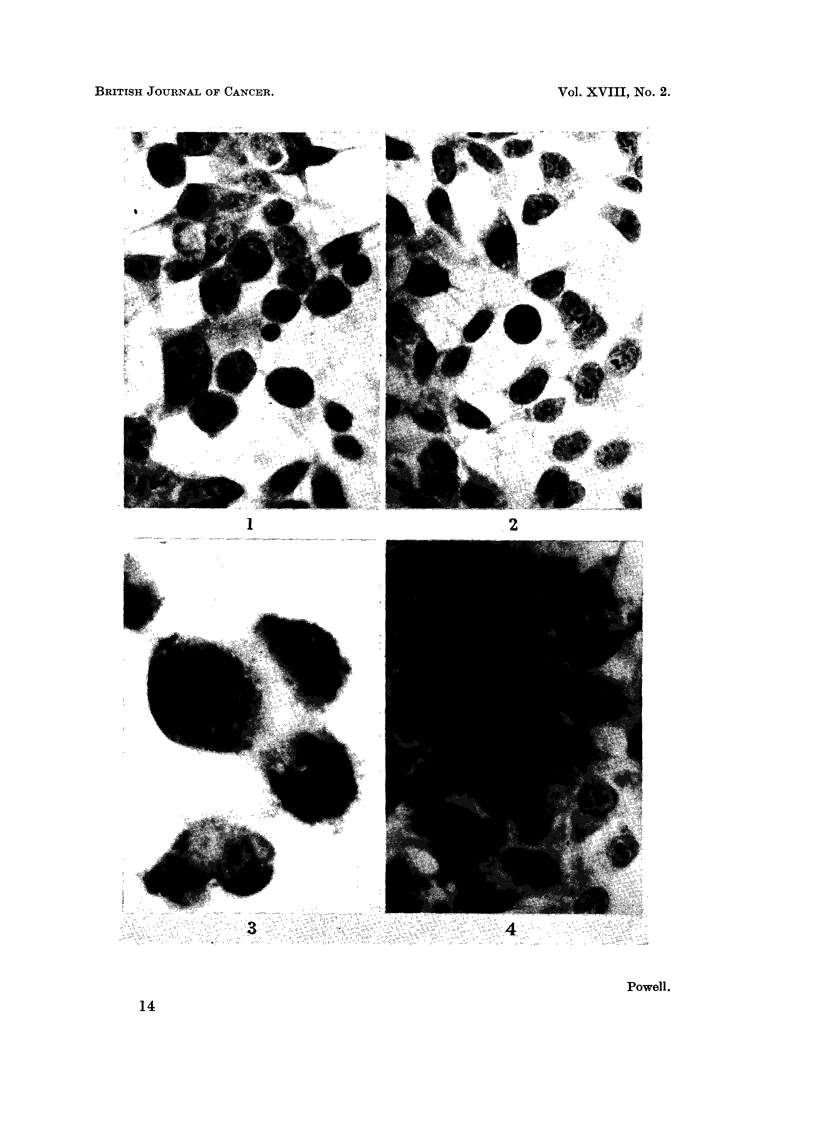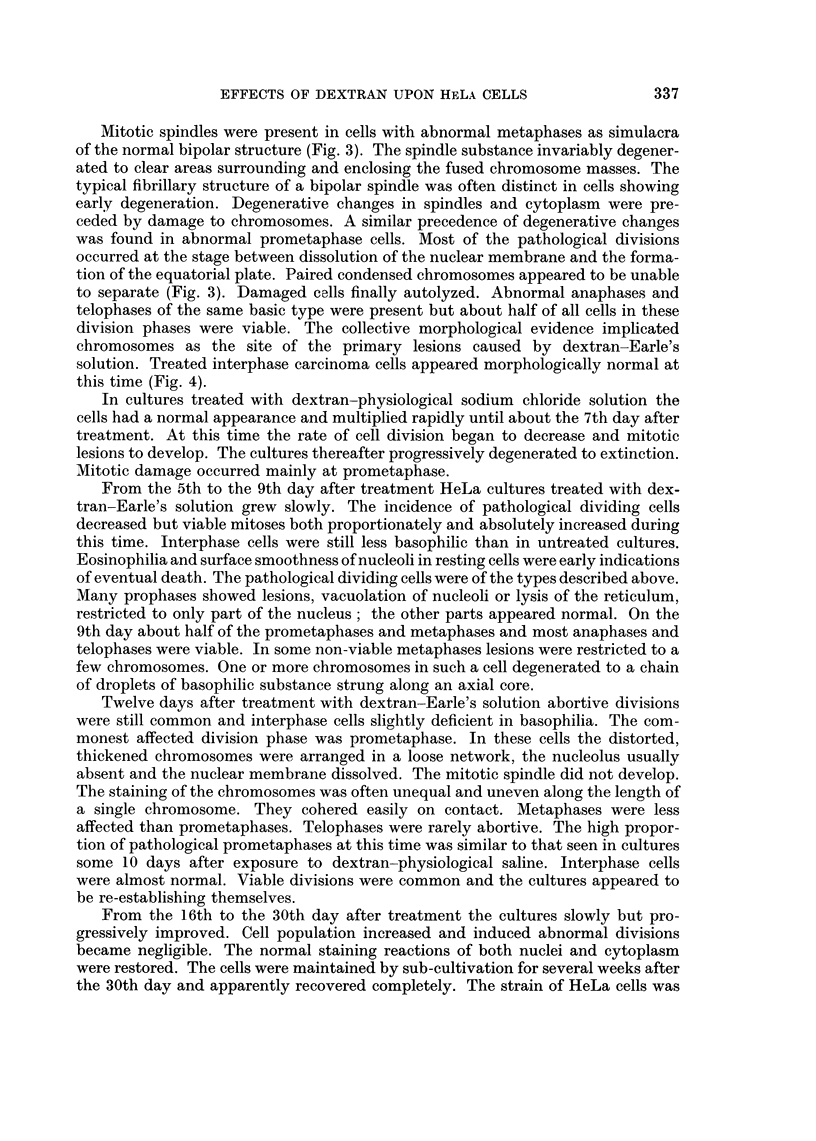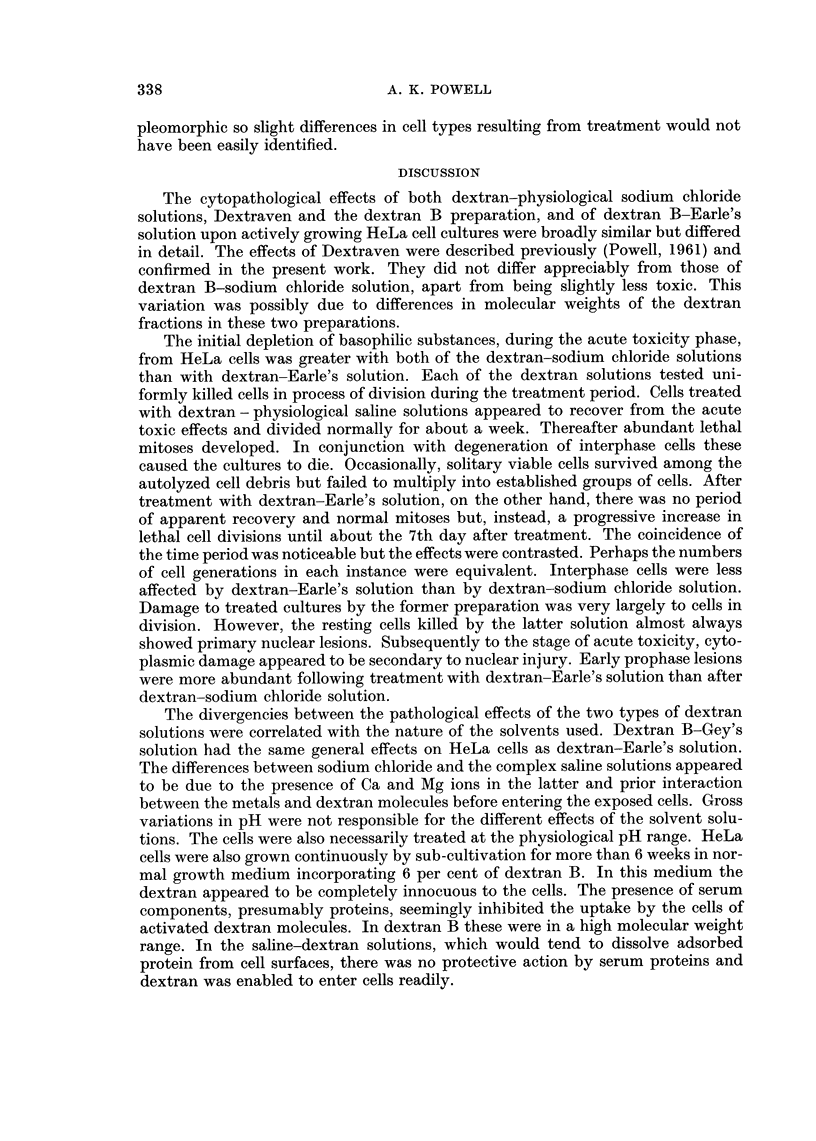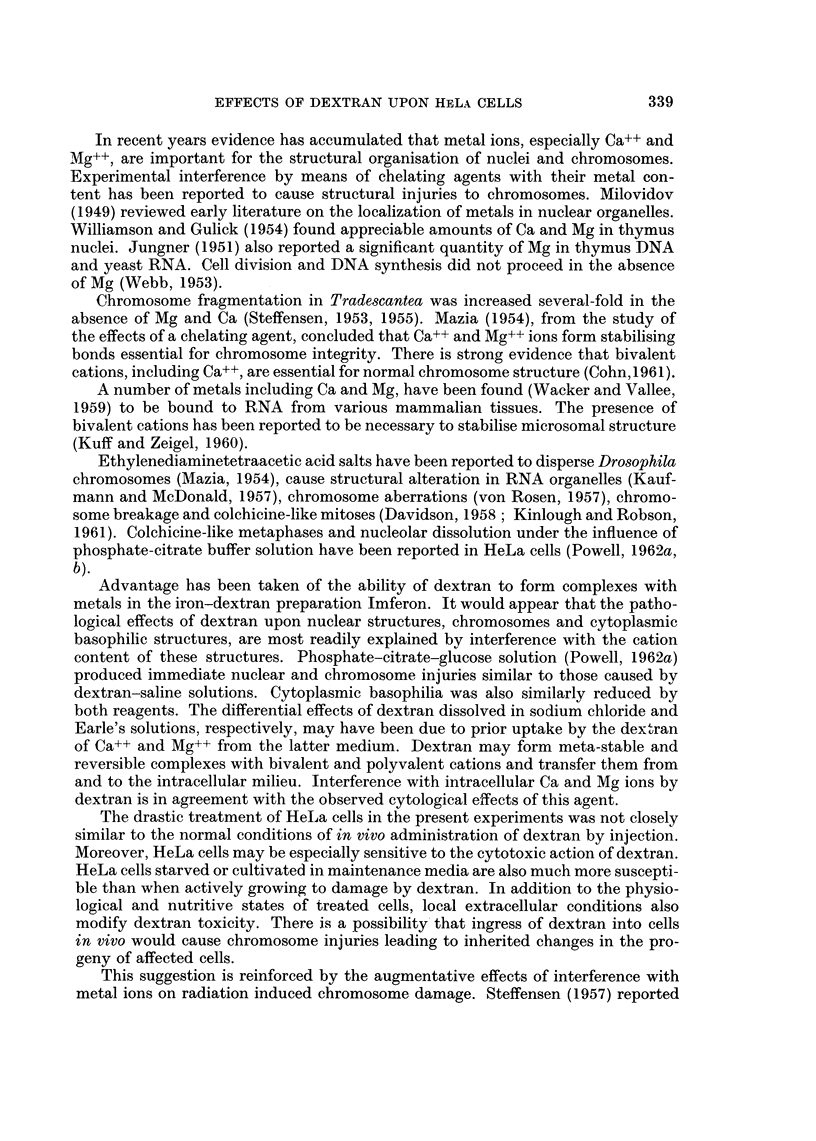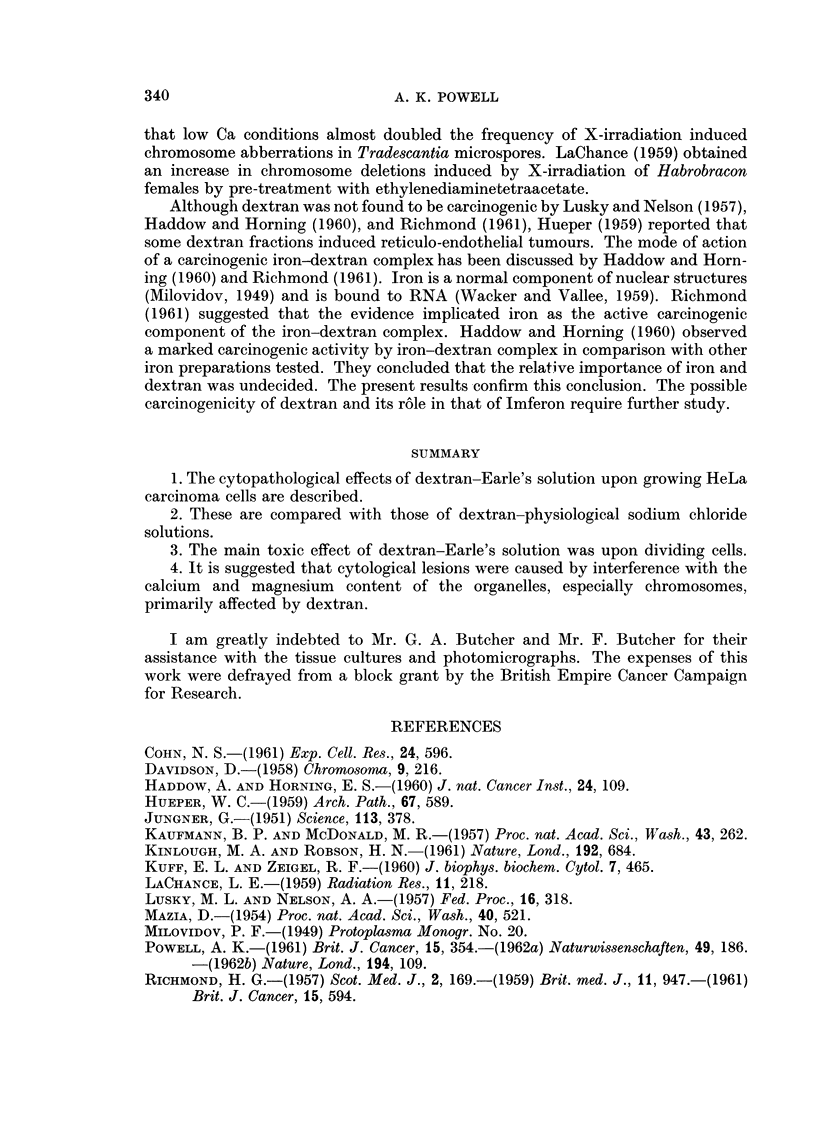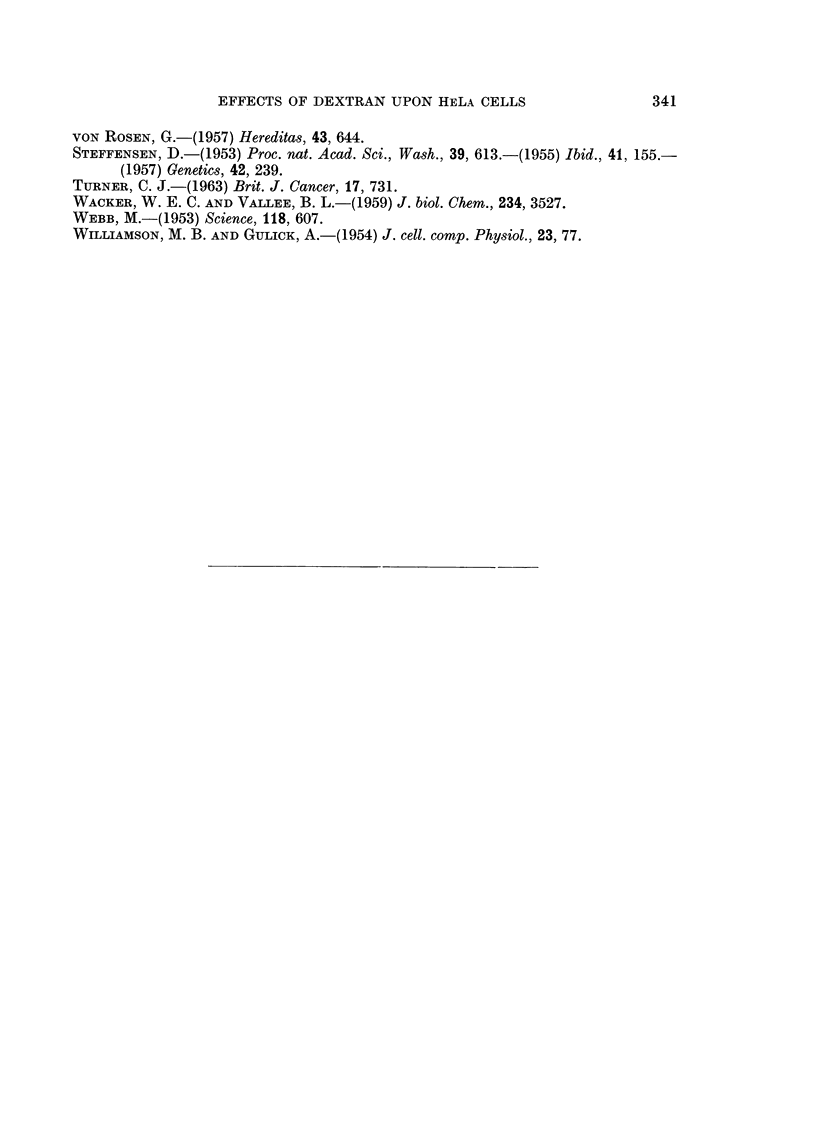# The Effects of Buffered Solutions of Dextran Upon HeLa Carcinoma Cells

**DOI:** 10.1038/bjc.1964.39

**Published:** 1964-06

**Authors:** A. K. Powell

## Abstract

**Images:**


### THE EFFECTS OF BUFFERED SOLUTIONS OF DEXTRAN UPON HELA CARCINOMA CELLS

A. K. POWELL

*From the Department of Experimental Pathology, Mount Vernon Hospital, Northwood, Middlesex*

Received for publication March 18, 1964

AN iron–dextran complex, Imferon, has been found to be carcinogenic to animals by Richmond (1957, 1959) and Haddow and Horning (1960). Dextran itself was reported to be non-carcinogenic by those workers and previously by Lusky and Nelson (1957). Richmond (1961) further adduced evidence that the carcinogenicity of Imferon is due to its iron content, the rôle of dextran being solely that of a vehicle. Hueper (1959), however, claimed an increased incidence of reticulo-endothelial tumours in rats following administration of dextran.

The effects of Imferon on several established lines of mammalian cells *in vitro* have been studied by Richmond (1961) and on cultivated chick embryo fibrocytes by Turner (1963). Both workers reported similarities between the effects of Imferon and those of known carcinogens in cultivated cells. In these experiments the iron–dextran complex was incorporated in culture media for administration to the cultured cells.

The effects of dextran–physiological saline solution (Dextraven, Benger Laboratories) upon actively growing HeLa carcinoma cells have been described previously (Powell, 1961). This preparation had an unusual effect upon the cultivated cells. After a rapid recovery from the initial toxicity of the dextran solution, HeLa cells grew normally for several days before showing a delayed but severe toxic effect. The treated cultures then rapidly degenerated. It has since been found that the cytotoxicity of dextran preparations is largely determined by the physico-chemical nature of the solvent used. The stage of apparent recovery after treatment of HeLa cells with dextran–sodium chloride solution was absent from cultures exposed to dextran balanced salt solutions. The effects of both types of dextran solutions were primarily upon chromosomes.

Dextran saline and dextran glucose preparations are used as blood plasma substitutes. Any cytotoxic effects of dextran therefore require consideration. The effects of dextran upon chromosomes described in the present report may be relevant to a possible rôle of dextran as a carcinogen *sui generis* and in combination with iron.

### MATERIALS AND METHODS

*HeLa carcinoma cell cultures.*—Stock cultures were maintained as described previously (Powell, 1961).

*Dextran solutions.*—Stock solutions of 12 per cent dextran and 1·8 per cent sodium chloride in re-distilled water were sterilised by autoclaving. Double-strength balanced saline solutions were sterilised by Seitz filtration. The experimental solutions were prepared by mixing equal volumes of the sterile dextran and the respective saline solutions. Control saline solutions were prepared by diluting the stock solutions with re-distilled water. In each experiment all test solutions were prepared from the same stock solutions. Earle's solution was selected for trial as an example of a well-buffered balanced solution. Results obtained with Gey's balanced saline paralleled those with Earle's solution.

*Experimental cultures.*—The procedure detailed earlier (Powell, 1961) was again followed. Cultures were given thorough preliminary washings with the test solutions, in order to remove normal culture medium.

In preliminary experiments three dextran fractions, A, B and C (British Drug Houses) at a concentration of 6 per cent in 0·9 per cent sodium chloride solution were compared with Dextraven for cytotoxicity to HeLa cells. Fraction B, with a range of molecular weights from 150,000 to 200,000, was slightly more toxic than Dextraven. Otherwise the effects of the two preparations did not differ appreciably. Dextran B was therefore used for the comparative study of the effects of dextran–physiological saline and dextran–Earle's solutions. The final concentration of dextran was 6 per cent in the experimental solutions. For the present experiments the duration of treatment was always 4 hours. This was near the optimum exposure for the demonstration of cytotoxicity and of cytological lesions without rapidly killing the cultures. After treatment the cultures were thoroughly rinsed and then fed with normal growth medium until the end of the experiments.

In each experiment sets of roller-tubes, usually 5–6 per set, were treated, respectively, with normal growth medium (controls), simple 0·9 per cent sodium chloride solution, 6 per cent dextran in 0·9 per cent sodium chloride solution, pure Earle's solution and 6 per cent of dextran in Earle's solution, respectively. Standard untreated control cultures were also used. Manipulation of cultures with ordinary medium had no perceptible effects. HeLa cells were also continuously cultivated on coverslips in roller-tubes in growth medium, incorporating 6 per cent of dextran B, for over 6 weeks. These treated cells were compared with collateral normal controls.

### EXPERIMENTAL RESULTS

The effects of Dextraven and 0·9 per cent sodium chloride solution used as control medium upon healthy and actively multiplying HeLa carcinoma cells have been described previously (Powell, 1961). The responses of HeLa cells to dextran B dissolved in physiological sodium chloride solution differed from those to Dextraven only in the slightly greater toxicity of the former preparation. The common effects of these dextran solutions will therefore be discussed only in reference to the effects of dextran B dissolved in Earle's saline solution.

The cytotoxic effects of Earle's solution itself were negligible. Immediately after exposure to this solution for 4 hours the HeLa cells had become more transparent and sharply defined but their basophilic content was unchanged. Normal cell divisions were present. The incidence of degenerating cells was perhaps slightly increased but this was not significant. The cultures grew rapidly and showed no ill effects of treatment.

Actively growing untreated HeLa carcinoma cell cultures invariably contained up to 5 per cent of degenerating and dead cells. Allowing for this minority of non-viable cells, in cultures fixed immediately after treatment with dextran dissolved in Earle's saline resting cells appeared to be almost unaffected. They were morphologically normal and healthy in appearance, apart from a slight but definite decrease in basophilia. This decrease was not enough to produce a compensating increase in acidophilia. It was greater in cytoplasm than nuclei ; neither nucleoli nor chromocentres showed any tendency to stain with eosin. Occasional interphase cells with almost normal nuclei but pathological cytoplasmic processes were present. These may have been sickly before treatment and thus more susceptible than healthy cells to injury.

Dextraven and dextran B–physiological sodium chloride solution caused a significantly greater immediate loss of basophilic substances than dextran–Earle's solution from interphase HeLa cells. Treatment with the former preparations, especially dextran B, tended to produce definite acidophilia in cytoplasm and nucleoli but chromocentres were usually still weakly basophilic in affected cells. The immediate effects of Earle's solution itself were slightly less evident than those of simple physiological saline.

The order of the severity of the immediate cytotoxicity of the two types of dextran preparations, physiological sodium chloride and buffered saline solutions respectively, found for interphase cells was reversed for dividing cells. The frequency of pyknotic dividing cells was much higher in the cultures treated with dextran–Earle's solution. This difference may have been due to less inhibition of mitosis by the more physiological Earle's solution. Cells at all phases of mitosis were irreversibly injured. There was no selective toxicity to any of the post-prophase division stages. This suggested that cells in post-prophase division phases when treatment began were rapidly damaged. Normal viable cell divisions were not found. The apparent absence of prophases indicated that this mitotic stage was not immediately injured and progressed to the dissolution of the nuclear membrane. The absence of the latter enabled the dextran to attack chromosomes rapidly. Injured metaphase cells showed a sequence of changes between early structural alterations, cohesion of damaged chromosomes, and, finally, coalescence into densely stained amorphous masses. This variation in effect also suggested that prophase cells tended to continue dividing in the test medium. Anaphase and telophase cells were usually pyknotic and presumably dividing at the beginning of treatment. Pyknotic dividing cells were usually characterised by increased eosinophilia and the development of numerous small irregular cytoplasmic extrusions.

There was no significant further increase in mortality among resting cells during the first day after treatment. They were viable but had become somewhat less basophilic than previously. Their cytoplasm retained its normal structure and nuclear lesions were inapparent. Dividing cells killed directly by exposure to the dextran–Earle's solution underwent further post-mortem changes.

Actively dividing HeLa cells were common. Normal viable divisions at all phases of mitosis were present but outnumbered by pathological divisions of all phases, including prophase. Pathological prophases and telophases were usually associated with peripheral cytoplasmic disintegration. The cytoplasm of these cells was often strongly eosinophilic. In later stages of degeneration of these cells the basophilic components of nuclear structures segregated into small droplets lying free from disrupted fragments of the intranuclear reticulum. Pathological prometaphases, metaphases and anaphases showed fragmentation and clumping of chromosomes and, finally, structural collapse of formed nuclear elements into separate basophilic and acidophilic droplets.

The effects of dextran–physiological sodium chloride solution on dividing cells at this time were much less drastic. Few mitoses were pathological. Recent pyknotic chromosome masses were almost absent and damaged prophases not found. Dextran–Earle's solution was more injurious than dextran–physiological saline although Earle's solution itself was less harmful than pure sodium chloride solution to HeLa cells. There was no selective effect at this time on any individual phase of mitosis by dextran–Earle's solution. Some cells divided successfully as far as telophase while others died in prophase. This wide spectrum of mitotic damage was probably due to varying severity of initial damage.

Two days after treatment interphase HeLa cells appeared relatively normal in morphology and cytoplasmic texture. Their basophilic content was only slightly subnormal. The incidence of dividing cells was less than at 24 hours after treatment. The great majority of prometaphases and metaphases were of the pathological type described above. Less than 10 per cent of metaphases were viable. Abnormal anaphases and telophases were present but most cells in these mitotic phases were viable. Some pathological prophases were observed but were relatively uncommon. Mitotic injury was predominantly at prometaphase and metaphase when chromosomes were fully formed. Cultures exposed to dextran–physiological saline had seemingly recovered 48 hours after treatment and were dividing normally

On the 3rd and 4th days after treatment with dextran–Earle's solution the cultures continued to grow. The general appearances of the cultures remained unchanged except for the increasing percentage of cells with abortive prometaphases and metaphases. These accumulated. By the 5th day after treatment the very high incidence of pathological divisions was immediately apparent (Fig. 1, 2). In unselected microscope fields it was found that cells with pathological fused prometaphases and metaphases (Fig. 1, 3) formed 11·1 per cent of the total cell population (288 cells). Of 260 prometaphase and metaphase cells studied with a 1/12th oil immersion objective only 2·2 per cent were identified as normal. 81·4 per cent were abnormal and the remaining 16·4 per cent probably but not certainly non-viable. These approximate incidences sufficed to indicate the magnitude of the effects of dextran–Earle's solution upon HeLa cell cultures.

All these lesions appeared to be caused by structural dissolution of chromosomes and chromonemata. Prophases showed considerable injuries. In these the basophilic material of the chromocentres in particular, but also of the nucleoli, separated into discrete droplets which became globular as the nuclear reticulum collapsed. In degenerated prometaphases and metaphases the outer basophilic material of the chromosomes formed beaded droplets along the axial chromosome threads and fused on contact with each other. Irregular and heterogeneous basophilic masses formed and later became spheroidal with clear droplets embedded in a basophilic matrix. Droplets of acidophilic material were often segregated from the basophilic material.

---

### EXPLANATION OF PLATE

FIG. 1.—HeLa carcinoma cells 5 days after treatment with dextran–Earle's solution, showing high incidence of pathological mitoses.

FIG. 2.—Untreated HeLa cells. Control to Fig. 1.

FIG. 3.—Pathological metaphase induced by dextran–Earle's solution 5 days after treatment. Shrunken paired chromosomes have failed to separate. Other chromosomes have fused to form pyknotic masses and the mitotic spindle is degenerate.

FIG. 4.—Morphologically viable interphase HeLa cells 5 days after treatment with dextran–Earle's solution.

Powell.

Mitotic spindles were present in cells with abnormal metaphases as simulacra of the normal bipolar structure (Fig. 3). The spindle substance invariably degenerated to clear areas surrounding and enclosing the fused chromosome masses. The typical fibrillary structure of a bipolar spindle was often distinct in cells showing early degeneration. Degenerative changes in spindles and cytoplasm were preceded by damage to chromosomes. A similar precedence of degenerative changes was found in abnormal prometaphase cells. Most of the pathological divisions occurred at the stage between dissolution of the nuclear membrane and the formation of the equatorial plate. Paired condensed chromosomes appeared to be unable to separate (Fig. 3). Damaged cells finally autolyzed. Abnormal anaphases and telophases of the same basic type were present but about half of all cells in these division phases were viable. The collective morphological evidence implicated chromosomes as the site of the primary lesions caused by dextran–Earle's solution. Treated interphase carcinoma cells appeared morphologically normal at this time (Fig. 4).

In cultures treated with dextran–physiological sodium chloride solution the cells had a normal appearance and multiplied rapidly until about the 7th day after treatment. At this time the rate of cell division began to decrease and mitotic lesions to develop. The cultures thereafter progressively degenerated to extinction. Mitotic damage occurred mainly at prometaphase.

From the 5th to the 9th day after treatment HeLa cultures treated with dextran–Earle's solution grew slowly. The incidence of pathological dividing cells decreased but viable mitoses both proportionately and absolutely increased during this time. Interphase cells were still less basophilic than in untreated cultures. Eosinophilia and surface smoothness of nucleoli in resting cells were early indications of eventual death. The pathological dividing cells were of the types described above. Many prophases showed lesions, vacuolation of nucleoli or lysis of the reticulum, restricted to only part of the nucleus ; the other parts appeared normal. On the 9th day about half of the prometaphases and metaphases and most anaphases and telophases were viable. In some non-viable metaphases lesions were restricted to a few chromosomes. One or more chromosomes in such a cell degenerated to a chain of droplets of basophilic substance strung along an axial core.

Twelve days after treatment with dextran–Earle's solution abortive divisions were still common and interphase cells slightly deficient in basophilia. The commonest affected division phase was prometaphase. In these cells the distorted, thickened chromosomes were arranged in a loose network, the nucleolus usually absent and the nuclear membrane dissolved. The mitotic spindle did not develop. The staining of the chromosomes was often unequal and uneven along the length of a single chromosome. They cohered easily on contact. Metaphases were less affected than prometaphases. Telophases were rarely abortive. The high proportion of pathological prometaphases at this time was similar to that seen in cultures some 10 days after exposure to dextran–physiological saline. Interphase cells were almost normal. Viable divisions were common and the cultures appeared to be re-establishing themselves.

From the 16th to the 30th day after treatment the cultures slowly but progressively improved. Cell population increased and induced abnormal divisions became negligible. The normal staining reactions of both nuclei and cytoplasm were restored. The cells were maintained by sub-cultivation for several weeks after the 30th day and apparently recovered completely. The strain of HeLa cells was pleomorphic so slight differences in cell types resulting from treatment would not have been easily identified.

### DISCUSSION

The cytopathological effects of both dextran–physiological sodium chloride solutions, Dextraven and the dextran B preparation, and of dextran B–Earle's solution upon actively growing HeLa cell cultures were broadly similar but differed in detail. The effects of Dextraven were described previously (Powell, 1961) and confirmed in the present work. They did not differ appreciably from those of dextran B–sodium chloride solution, apart from being slightly less toxic. This variation was possibly due to differences in molecular weights of the dextran fractions in these two preparations.

The initial depletion of basophilic substances, during the acute toxicity phase, from HeLa cells was greater with both of the dextran–sodium chloride solutions than with dextran–Earle's solution. Each of the dextran solutions tested uniformly killed cells in process of division during the treatment period. Cells treated with dextran – physiological saline solutions appeared to recover from the acute toxic effects and divided normally for about a week. Thereafter abundant lethal mitoses developed. In conjunction with degeneration of interphase cells these caused the cultures to die. Occasionally, solitary viable cells survived among the autolyzed cell debris but failed to multiply into established groups of cells. After treatment with dextran–Earle's solution, on the other hand, there was no period of apparent recovery and normal mitoses but, instead, a progressive increase in lethal cell divisions until about the 7th day after treatment. The coincidence of the time period was noticeable but the effects were contrasted. Perhaps the numbers of cell generations in each instance were equivalent. Interphase cells were less affected by dextran–Earle's solution than by dextran–sodium chloride solution. Damage to treated cultures by the former preparation was very largely to cells in division. However, the resting cells killed by the latter solution almost always showed primary nuclear lesions. Subsequently to the stage of acute toxicity, cytoplasmic damage appeared to be secondary to nuclear injury. Early prophase lesions were more abundant following treatment with dextran–Earle's solution than after dextran–sodium chloride solution.

The divergencies between the pathological effects of the two types of dextran solutions were correlated with the nature of the solvents used. Dextran B–Gey's solution had the same general effects on HeLa cells as dextran–Earle's solution. The differences between sodium chloride and the complex saline solutions appeared to be due to the presence of Ca and Mg ions in the latter and prior interaction between the metals and dextran molecules before entering the exposed cells. Gross variations in pH were not responsible for the different effects of the solvent solutions. The cells were also necessarily treated at the physiological pH range. HeLa cells were also grown continuously by sub-cultivation for more than 6 weeks in normal growth medium incorporating 6 per cent of dextran B. In this medium the dextran appeared to be completely innocuous to the cells. The presence of serum components, presumably proteins, seemingly inhibited the uptake by the cells of activated dextran molecules. In dextran B these were in a high molecular weight range. In the saline–dextran solutions, which would tend to dissolve adsorbed protein from cell surfaces, there was no protective action by serum proteins and dextran was enabled to enter cells readily.

In recent years evidence has accumulated that metal ions, especially $Ca^{++}$ and $Mg^{++}$, are important for the structural organisation of nuclei and chromosomes. Experimental interference by means of chelating agents with their metal content has been reported to cause structural injuries to chromosomes. Milovidov (1949) reviewed early literature on the localization of metals in nuclear organelles. Williamson and Gulick (1954) found appreciable amounts of Ca and Mg in thymus nuclei. Jungner (1951) also reported a significant quantity of Mg in thymus DNA and yeast RNA. Cell division and DNA synthesis did not proceed in the absence of Mg (Webb, 1953).

Chromosome fragmentation in *Tradescantea* was increased several-fold in the absence of Mg and Ca (Steffensen, 1953, 1955). Mazia (1954), from the study of the effects of a chelating agent, concluded that $Ca^{++}$ and $Mg^{++}$ ions form stabilising bonds essential for chromosome integrity. There is strong evidence that bivalent cations, including $Ca^{++}$, are essential for normal chromosome structure (Cohn, 1961).

A number of metals including Ca and Mg, have been found (Wacker and Vallee, 1959) to be bound to RNA from various mammalian tissues. The presence of bivalent cations has been reported to be necessary to stabilise microsomal structure (Kuff and Zeigel, 1960).

Ethylenediaminetetraacetic acid salts have been reported to disperse *Drosophila* chromosomes (Mazia, 1954), cause structural alteration in RNA organelles (Kaufmann and McDonald, 1957), chromosome aberrations (von Rosen, 1957), chromosome breakage and colchicine-like mitoses (Davidson, 1958 ; Kinlough and Robson, 1961). Colchicine-like metaphases and nucleolar dissolution under the influence of phosphate-citrate buffer solution have been reported in HeLa cells (Powell, 1962*a*, *b*).

Advantage has been taken of the ability of dextran to form complexes with metals in the iron–dextran preparation Imferon. It would appear that the pathological effects of dextran upon nuclear structures, chromosomes and cytoplasmic basophilic structures, are most readily explained by interference with the cation content of these structures. Phosphate–citrate–glucose solution (Powell, 1962*a*) produced immediate nuclear and chromosome injuries similar to those caused by dextran–saline solutions. Cytoplasmic basophilia was also similarly reduced by both reagents. The differential effects of dextran dissolved in sodium chloride and Earle's solutions, respectively, may have been due to prior uptake by the dextran of $Ca^{++}$ and $Mg^{++}$ from the latter medium. Dextran may form meta-stable and reversible complexes with bivalent and polyvalent cations and transfer them from and to the intracellular milieu. Interference with intracellular Ca and Mg ions by dextran is in agreement with the observed cytological effects of this agent.

The drastic treatment of HeLa cells in the present experiments was not closely similar to the normal conditions of *in vivo* administration of dextran by injection. Moreover, HeLa cells may be especially sensitive to the cytotoxic action of dextran. HeLa cells starved or cultivated in maintenance media are also much more susceptible than when actively growing to damage by dextran. In addition to the physiological and nutritive states of treated cells, local extracellular conditions also modify dextran toxicity. There is a possibility that ingress of dextran into cells *in vivo* would cause chromosome injuries leading to inherited changes in the progeny of affected cells.

This suggestion is reinforced by the augmentative effects of interference with metal ions on radiation induced chromosome damage. Steffensen (1957) reported that low Ca conditions almost doubled the frequency of X-irradiation induced chromosome abberrations in *Tradescantia* microspores. LaChance (1959) obtained an increase in chromosome deletions induced by X-irradiation of *Habrobracon* females by pre-treatment with ethylenediaminetetraacetate.

Although dextran was not found to be carcinogenic by Lusky and Nelson (1957), Haddow and Horning (1960), and Richmond (1961), Hueper (1959) reported that some dextran fractions induced reticulo-endothelial tumours. The mode of action of a carcinogenic iron–dextran complex has been discussed by Haddow and Horning (1960) and Richmond (1961). Iron is a normal component of nuclear structures (Milovidov, 1949) and is bound to RNA (Wacker and Vallee, 1959). Richmond (1961) suggested that the evidence implicated iron as the active carcinogenic component of the iron–dextran complex. Haddow and Horning (1960) observed a marked carcinogenic activity by iron–dextran complex in comparison with other iron preparations tested. They concluded that the relative importance of iron and dextran was undecided. The present results confirm this conclusion. The possible carcinogenicity of dextran and its rôle in that of Imferon require further study.

### SUMMARY

1. The cytopathological effects of dextran–Earle's solution upon growing HeLa carcinoma cells are described.

2. These are compared with those of dextran–physiological sodium chloride solutions.

3. The main toxic effect of dextran–Earle's solution was upon dividing cells.

4. It is suggested that cytological lesions were caused by interference with the calcium and magnesium content of the organelles, especially chromosomes, primarily affected by dextran.

I am greatly indebted to Mr. G. A. Butcher and Mr. F. Butcher for their assistance with the tissue cultures and photomicrographs. The expenses of this work were defrayed from a block grant by the British Empire Cancer Campaign for Research.

### REFERENCES

Cohn, N. S.—(1961) *Exp. Cell. Res.*, **24**, 596.
Davidson, D.—(1958) *Chromosoma*, **9**, 216.
Haddow, A. and Horning, E. S.—(1960) *J. nat. Cancer Inst.*, **24**, 109.
Hueper, W. C.—(1959) *Arch. Path.*, **67**, 589.
Jungner, G.—(1951) *Science*, **113**, 378.
Kaufmann, B. P. and McDonald, M. R.—(1957) *Proc. nat. Acad. Sci., Wash.*, **43**, 262.
Kinlough, M. A. and Robson, H. N.—(1961) *Nature, Lond.*, **192**, 684.
Kuff, E. L. and Zeigel, R. F.—(1960) *J. biophys. biochem. Cytol.* **7**, 465.
LaChance, L. E.—(1959) *Radiation Res.*, **11**, 218.
Lusky, M. L. and Nelson, A. A.—(1957) *Fed. Proc.*, **16**, 318.
Mazia, D.—(1954) *Proc. nat. Acad. Sci., Wash.*, **40**, 521.
Milovidov, P. F.—(1949) *Protoplasma Monogr.* No. 20.
Powell, A. K.—(1961) *Brit. J. Cancer*, **15**, 354.—(1962*a*) *Naturwissenschaften*, **49**, 186.—(1962*b*) *Nature, Lond.*, **194**, 109.
Richmond, H. G.—(1957) *Scot. Med. J.*, **2**, 169.—(1959) *Brit. med. J.*, **11**, 947.—(1961) *Brit. J. Cancer*, **15**, 594.

von Rosen, G.—(1957) *Hereditas*, **43**, 644.
Steffensen, D.—(1953) *Proc. nat. Acad. Sci., Wash.*, **39**, 613.—(1955) *Ibid.*, **41**, 155.—(1957) *Genetics*, **42**, 239.
Turner, C. J.—(1963) *Brit. J. Cancer*, **17**, 731.
Wacker, W. E. C. and Vallee, B. L.—(1959) *J. biol. Chem.*, **234**, 3527.
Webb, M.—(1953) *Science*, **118**, 607.
Williamson, M. B. and Gulick, A.—(1954) *J. cell. comp. Physiol.*, **23**, 77.